# Bouveret's Syndrome: Case Report and Review of the Literature

**DOI:** 10.1155/2009/914951

**Published:** 2009-04-07

**Authors:** Iliana Doycheva, Alpna Limaye, Amitabh Suman, Christopher E. Forsmark, Shahnaz Sultan

**Affiliations:** ^1^Division of Gastroenterology, Hepatology, and Nutrition, College of Medicine, University of Florida, Gainesville, FL 32611, USA; ^2^Division of Gastroenterology, Hepatology, and Nutrition, Malcom Randall Veterans Affairs Medical Center, College of Medicine, University of Florida, Gainesville, FL 32608, USA; ^3^Division of Gastroenterology, Hepatology, and Nutrition, Malcom Randall Veterans Affairs Medical Center, Rehabilitation Outcomes Research Center, College of Medicine, University of Florida, Gainesville, FL 32608, USA

## Abstract

Bouveret's syndrome is defined as gastric outlet obstruction caused by duodenal impaction of a large gallstone which passes into the duodenal bulb through a cholecystogastric or cholecystoduodenal fistula. Initial attempts at endoscopic retrieval with or without mechanical or extracorporeal lithotripsy should be performed as first-line treatment, though success rates with endoscopic treatment are variable. We describe a case of Bouveret's Syndrome in an elderly patient that was successfully treated with endoscopic extraction combined with mechanical lithotripsy, and review the literature on this uncommon condition.

## 1. Introduction

The first published report of Bouveret's syndrome (1896) is attributed to Leon Bouveret who reported on two patients with this disease [1]. Since then, there have been several case reports of unique manifestations of Bouveret's syndrome, as well as reports of novel endoscopic treatment modalities. Bouveret's syndrome tends to occur more commonly in women (65%) with a median age of 74.1 years at presentation [2]. Because it often presents in patients with advanced age and multiple comorbidities, it is associated with a high rate of mortality. Therefore, endoscopic treatment should always be attempted in order to avoid surgery in these patients. For patients in whom endoscopic extraction has failed, simple enterolithotomy, duodenotomy or gastrotomy, and stone extraction can be performed [3]. 

## 2. Case Presentation

### 2.1. History

An 86-year-old Caucasian male was admitted with a three-day history of nausea, vomiting, left lower quadrant abdominal pain and three episodes of melena. He had not consumed any food for the previous two days. He had a past medical history significant for bladder cancer, hypertension, peripheral vascular disease, and gallstones. 

Two months prior to this presentation, the patient had been admitted with elevated liver function tests. Abdominal computed tomography (CT) revealed gallbladder thickening with surrounding stranding, cholelithiasis, and choledocholithiasis with a 7 mm stone in the common bile duct. On endoscopic retrograde cholangiopancreatography, a sphincterotomy was performed, three gallstones (the largest of which was 12 mm in diameter) were removed from the common bile duct, and a biliary stent was left in place. There were no complications and the patient was well until he presented two months later.

### 2.2. Exam

**Figure 1:**
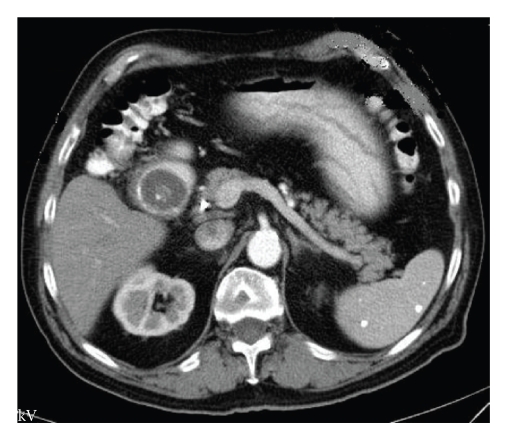
A gallstone in the duodenum and a distended stomach was seen on CT scan.

On physical exam, patient was in no acute distress, afebrile, and hemodynamically stable. Pertinent findings included mild tenderness to palpation in the epigastric area, an audible succession splash and normal bowel sounds. His liver enzymes, electrolytes, and creatinine level were all within normal limits. CT of the abdomen revealed pneumobilia, the presence of a biliary stent, a distended stomach and a 3.3 cm hypodense oval object in the second portion of the duodenum suggestive of Bouveret's Syndrome (Figure 1). 

### 2.3. Management

**Figure 2:**
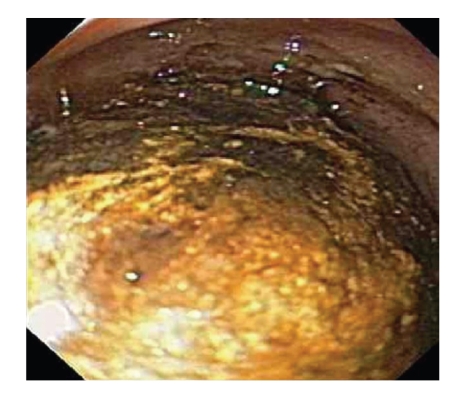
Endoscopic image showing the gallstone causing obstruction.

**Figure 3:**
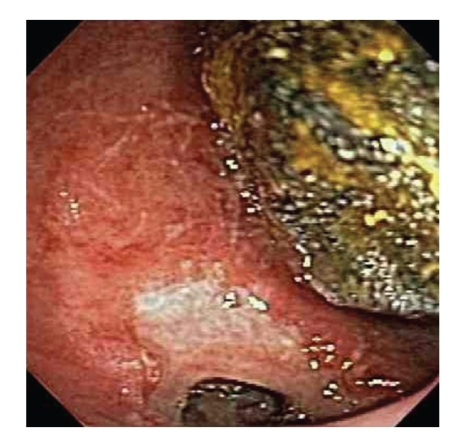
Fistula and surrounding ulceration of the duodenum on endoscopy.

Initial esophagogastroduodenoscopy (EGD) revealed large amounts of fluid and food content in the stomach with poor visualization of the duodenum due to retained food and a large stone in the second part of duodenum causing complete obstruction of its lumen (Figure 2). Extensive ulceration of the duodenal wall at the point where the stone was impacted was also seen. The patient was brought back the following day for attempted stone extraction, at which time the stone was successfully removed from the duodenum and transferred to the stomach using various devices including a retrieval net, snare, stone extracting basket, and lithotripsy basket. A cholecystoduodenal fistula was visualized beneath the level of the stone on subsequent endoscopy (Figure 3). The stone was then successfully crushed into smaller fragments using a mechanical lithotripter. After successful endoscopic treatment, the patient's symptoms resolved and he was discharged home with close follow-up as an outpatient.

## 3. Discussion

Cholelithiasis is a relatively common health problem; by age 75, about 35% of women and 20% of men have developed gallstones [4]. While the majority of patients with gallstones do well, a small percentage of patients (approximately 6%) develop complications including such rare complications as cholecystoduodenal fistulas [5]. Fistula formation is thought to occur as a result of adhesions between the gallbladder and the bowel wall from chronic inflammation, impaired arterial blood supply and decreased venous drainage [6]. Ensuing fistula formation can occur from pressure necrosis and compression of a gallstone against the gallbladder wall, and subsequent passage of gallstones via the fistula can then result in *gallstone ileus* [7]. 

The most common location where a gallstone gets “stuck” is in the ileum (84%), usually in the terminal portion. In 1–3% of cases, however, the gallstone gets lodged in the duodenum and causes symptoms consistent with gastric outlet obstruction; it is this rare condition that is known as *Bouveret's Syndrome* [8]. The syndrome was first described by Beaussier in 1770 and was subsequently named after the French physician Leon Bouveret after he published two case reports in Revue de Medecin in 1896 [1]. 

### 3.1. Diagnosis

In most cases, the presenting signs and symptoms of Bouveret's syndrome are nonspecific. In a recent systematic review, Cappell and Davis described the most common symptoms of patients with Bouveret's syndrome as nausea and vomiting (86%), and abdominal pain (71%); less commonly, patients present with hematemesis, weight loss, and anorexia [2]. On physical exam, patients often have abdominal tenderness, abdominal distention, and dehydration. 

The diagnosis of Bouveret's syndrome is usually made via endoscopy. Grove, in 1976, was the first to describe a case of pyloric obstruction due to a gallstone as diagnosed by gastroscopy [9]. On endoscopy, visualization of a stone causing obstruction is seen in about 69% of patients and obstruction in the absence of a visualized stone or fistula is seen in 31% of cases [2]. In the remaining cases, the stone may not actually be visualized because it is compressing the lumen and only partially visualized through the duodenal wall. Other findings described include excessive retained food or fluid in the stomach and inflammation, edema, or ulcer at the impacted site [2]. 

Radiologic imaging tests can often confirm the diagnosis of Bouveret's syndrome. CT is diagnostic in about 60% of cases and is helpful in demonstrating the exact level of obstruction, the biliary site of the duodenal fistula, and the status of the gallbladder [10, 11]. Approximately 15–25% of gallstones are isoattenuating and not well visualized on CT. In such cases, magnetic resonance cholangiopancreatography (MRCP) may be useful, because it may more clearly delineate fluid from calculi.

### 3.2. Treatment

Endoscopic extraction, endoscopic laser lithotripsy (ILL), extracorporeal shockwave lithotripsy (ESWL), and intracorporeal electrohydraulic lithotripsy (IEHL) have all been reported as alternatives to surgery for more proximal gallstone obstruction whereas surgery is routinely recommended for individuals with impaction of the gallstone more distally (gallstone ileus). 

Endoscopic treatment of Bouveret's syndrome should be considered a first-line option despite the low success rate reported in the literature. The first successful endoscopic extraction was described in 1985 by Bedogni et al. [12]. Subsequently, a number of case reports have been published describing successful endoscopic management of Bouveret's syndrome [3, 6–14]. Endoscopic management often necessitates the use of different sized and shaped snares, grasping forceps, retrieval baskets and nets, biliary balloons, and sometimes even a side-viewing endoscope; it can be technically challenging, time-consuming, and success rates in case series have been previously reportedly to be less than 10% [3]. 

Endoscopic treatment, accompanied by lithotripsy using a variety of different modalities, has been well described. There are several reports of intracorporeal laser lithotripsy (ILL) alone, and in combination with, ESWL [6, 14–16]. The first reported successful use of ILL was in 1999 by Maiss et al. requiring a total of nine sessions [14]. The drawbacks of this particular procedure are the need for prolonged and multiple sessions, and the risk of converting a proximal gallstone ileus into a distal gallstone ileus (as a result of partial fragmentation of the stone). 

IEHL can be used alone or in combination with other methods. Moriai et al. used IEHL with mechanical lithotripsy for removal of two 3 cm stones in 1991 [17]. In 1997 Dumonceau et al. reported a case of successful treatment with IEHL after failure of ESWL [15]. Two additional cases described by Huebner et al., in 2007 saw the incorporation of IEHL as the sole modality with successful stone extraction [18]. The risk involved with this method is that inadvertent focusing of the shockwaves onto the intestine wall may cause bleeding and perforation. 

Extracorporeal shockwave lithotripsy (ESWL) has also been used with success in treating patients with Bouveret's syndrome [15, 19–21]. Limitations of using ESWL include the need for several return sessions, in addition to eventual endoscopy. Also, ESWL may be difficult to perform in obese patients or if there are gas-containing bowel loops interposed between the gallstone and the abdominal wall [15, 19–21]. 

In general, the success rate of endoscopic extraction is dependent on stone size. Stones, that are larger than 2.5 cm are more difficult to extract endoscopically, although extractions of stones up to 3 cm, have been reported [3]. Larger stones can cause ischemic ulceration of the adjacent duodenal wall. Moreover, these stones tend to have a hard outer shell and soft inner core making mechanical fragmentation with endoscopic forceps or laser more difficult. While the majority of patients tolerate attempted endoscopic treatment, there has been a case report of pulseless electrical activity (PEA) during mechanical retrieval due to the gallstone getting lodged in the esophagus; the PEA abruptly resolved when the stone was pushed back into the stomach. In a handful of cases, surgery was needed subsequent to upper endoscopy due to stricture, sepsis, and a second stone in the duodenum [13]. 

If endoscopic treatment fails, the patient will require surgical management. Forty-two percent of surgical patients have previously undergone a failed endoscopic treatment. Surgical options include a combination of enterolithotomy (removal of the stone) plus cholecystectomy and fistula repair, but surgery is associated with significant morbidity and mortality [22–26]. Combined treatment has been associated with a higher mortality of 20–30%, compared to just 12% in cases of simple duodenotomy. Laparoscopy is also an additional option for surgical treatment; Sica et al. reported, in 2005, the first case of uneventful stone removal and cholecystectomy by laparoscopy [26]. 

Although debatable, fistula repair in patients treated with endoscopic methods or simple enterolithotomy is often considered unnecessary due to spontaneous closure, especially when the cystic duct is patent and no residual stones are present. On the other hand, the persistence of symptoms, the possibility for recurrence, and the risk for gallbladder cancer lend support to fistula repair.

## 4. Conclusions

Prior case series have reported low success rates with endoscopic treatment (9%) however it is likely that endoscopic success rates are much higher now, as more innovative endoscopic techniques are used to treat these patients. The use of mechanical and intracorporeal lithitripsy allows larger stones to be managed, and should be considered the first-line of therapy for this rare condition.
